# Factors influencing quality of life in patients with lower extremity varicose veins: a cross-sectional study

**DOI:** 10.3389/fmed.2026.1848120

**Published:** 2026-06-15

**Authors:** Liqin Wang, Yan Lin, Qiuni Cai, Fengxiong Wang

**Affiliations:** 1 Department of Nephrology, Zhongshan Hospital of Xiamen University, School of Medicine, Xiamen University, Xiamen, China; 2 Department of Rare Diseases, Zhongshan Hospital of Xiamen University, School of Medicine, Xiamen University, Xiamen, China; 3 Department of General Surgery, Zhongshan Hospital of Xiamen University, School of Medicine, Xiamen University, Xiamen, China; 4 Department of Foot and Ankle & Wound Healing, The First Affiliated Hospital of Guangdong Pharmaceutical University, Guangzhou, China

**Keywords:** Aberdeen Varicose Vein Questionnaire, chronic venous disease, quality of life, varicose veins, Venous Clinical Severity Score

## Abstract

**Background:**

Lower extremity varicose veins (LEVV) significantly impair health-related quality of life (HRQoL), but comprehensive evidence on physiological and psychological determinants remains limited, especially in developing countries. This study aimed to identify independent predictors of disease-specific HRQoL in patients with primary LEVV.

**Methods:**

A cross-sectional study was conducted from December 2023 to April 2025 at a university-affiliated hospital in China, enrolling 256 patients with primary LEVV (CEAP C2–C6). HRQoL was assessed using the Aberdeen Varicose Vein Questionnaire (AVVQ). Clinical severity was measured by the Venous Clinical Severity Score (VCSS) and CEAP classification. Psychological status was evaluated using the Hospital Anxiety and Depression Scale (HADS). Stepwise multiple linear regression was performed to identify independent predictors of AVVQ scores.

**Results:**

The mean AVVQ score was 18.6 ± 9.4, indicating moderate QoL impairment. Eight variables emerged as independent predictors in the final model (adjusted *R*^2^ = 0.611, *p* < 0.001): higher VCSS (*β* = 0.312), higher HADS-Anxiety score (*β* = 0.224), advanced CEAP classification (*β* = 0.198), higher HADS-Depression score (*β* = 0.156), greater number of venous symptoms (*β* = 0.142), higher BMI (*β* = 0.128), absence of regular physical activity (*β* = −0.108), and bilateral involvement (*β* = 0.096).

**Conclusion:**

Quality of life in patients with lower extremity varicose veins is independently influenced by clinical severity, psychological distress (anxiety and depression), higher BMI, physical inactivity, symptom burden, and bilateral disease. These findings highlight the need for comprehensive management strategies that integrate psychological screening, weight control, and exercise prescription alongside conventional clinical care.

## 1 Introduction

Chronic venous disease (CVD) of the lower limbs is one of the most prevalent vascular disorders worldwide, with varicose veins representing its most common clinical manifestation (1). Epidemiological studies indicate that among the adult population in developed countries, 27% of sedentary workers are affected by varicose veins, while the prevalence rises to 36% among standing workers (2). In developing countries, this proportion shows an increasing trend. The disease is characterized by the dilation and tortuosity of superficial veins, primarily involving the great saphenous vein and small saphenous vein systems, resulting from venous valve insufficiency and sustained venous hypertension (3). According to the Clinical-Etiological-Anatomical-Pathophysiological (CEAP) classification system, varicose veins encompass a broad spectrum of clinical presentations, ranging from telangiectasias and reticular veins (C1) to active venous ulcers (C6) (4).

The pathophysiology of lower extremity varicose veins involves a complex interplay of genetic predisposition, structural alterations in the venous wall, valvular dysfunction, and inflammatory processes (5). Established risk factors for the development of varicose veins include advanced age, female sex, obesity, prolonged standing, multiparity, and family history of venous disease (6). If left untreated or inadequately managed, varicose veins may progress to more severe stages of chronic venous insufficiency (CVI), leading to complications such as edema, skin changes, lipodermatosclerosis, and venous ulceration, which impose a substantial burden on both patients and healthcare systems (7).

Beyond the well-documented hemodynamic and dermatological consequences, varicose veins significantly affect patients’ health-related quality of life (HRQoL) (8). Patients commonly report symptoms including leg heaviness, aching, swelling, cramps, itching, and restless legs, all of which can interfere with daily activities, occupational performance, and sleep quality (9). Furthermore, the cosmetic disfigurement associated with visible varicose veins may lead to psychological distress, reduced self-esteem, body image dissatisfaction, anxiety, and social avoidance, particularly among female patients (10, 11). Multiple studies have demonstrated that the impairment in HRQoL experienced by patients with varicose veins is comparable to that observed in other chronic diseases, such as diabetes, arthritis, and cardiovascular disease (12).

Previous studies have identified several factors that may influence HRQoL in patients with chronic venous disease, including disease severity classified according to the CEAP system, symptom burden, body mass index (BMI), comorbidities, treatment modalities, and sociodemographic characteristics (13). However, current evidence still presents certain limitations. Firstly, the majority of previous studies have focused on patients with chronic venous disease rather than specifically on those with lower extremity varicose veins. Secondly, most existing research has been conducted in populations from developed countries, while data from populations in developing countries remain limited (14). Moreover, many prior studies have primarily concentrated on the physical aspects of quality of life, with relatively less attention given to the psychological, social, and occupational domains (15). Therefore, this study aims to comprehensively investigate the physiological and psychological factors affecting HRQoL in patients with lower extremity varicose veins. A thorough understanding of these factors is crucial for developing targeted interventions and personalized management strategies, which may contribute to improving the health status and long-term outcomes of patients with this common and often underestimated condition.

## 2 Methods

### 2.1 Study design and setting

This was a cross-sectional observational study conducted in a university-affiliated hospital, from December 2023 to April 2025. The study aimed to investigate factors influencing HRQoL in patients with lower extremity varicose veins (LEVV). All participants provided written informed consent prior to enrollment. The reporting of this manuscript adheres to the requirements for cross-sectional studies outlined in the Strengthening the Reporting of Observational Studies in Epidemiology (STROBE) guidelines (16).

### 2.2 Study population

#### 2.2.1 Inclusion criteria

Patients were eligible for inclusion if they met all of the following criteria: (1) age ≥ 18 years; (2) clinical diagnosis of primary lower extremity varicose veins confirmed by duplex ultrasonography; (3) classified as C2–C6 according to the CEAP classification system; (4) ability to read, understand, and independently complete the study questionnaires; and (5) willingness to provide written informed consent.

#### 2.2.2 Exclusion criteria

Patients were excluded if they met any of the following criteria: (1) secondary varicose veins due to deep venous thrombosis, post-thrombotic syndrome, or extrinsic venous compression; (2) history of previous surgical or endovenous treatment for varicose veins; (3) presence of severe systemic diseases that could independently and substantially impair quality of life; (4) current pregnancy or within 6 months postpartum; (5) diagnosed psychiatric disorders that could interfere with questionnaire completion; and (6) refusal to provide written informed consent.

### 2.3 Sample size calculation

The sample size was estimated based on the requirements for multiple linear regression analysis. According to the commonly recommended guideline proposed by Green (17), a minimum sample size for testing individual predictors in a multiple regression model should be calculated as *N* ≥ 50 + 8 k, where k represents the number of independent variables. Considering an estimated 20 independent variables to be included in the regression model, a minimum of 210 participants was required.

### 2.4 Data collection

Data were collected by trained research assistants through face-to-face interviews combined with self-administered questionnaires and clinical examinations. All data were collected at a single time point during the patients’ outpatient visit or hospital admission. The following categories of variables were assessed:

#### 2.4.1 Sociodemographic characteristics

Sociodemographic data were collected using a standardized data collection form, including age, sex, BMI, marital status (married, single, or divorced), educational level (primary school or below, secondary/high school, college/university or above), employment status (employed, unemployed, retired), occupation type (sedentary, prolonged standing, mixed), monthly household income, smoking status (current smoker, former smoker, non-smoker), alcohol consumption (yes/no), and regular physical activity (defined as at least 150 min of moderate-intensity aerobic exercise per week according to World Health Organization recommendations) (18).

#### 2.4.2 Clinical characteristics

Clinical data were obtained from medical records and physical examination, including: (1) duration of varicose veins (in years); (2) laterality of involvement (unilateral or bilateral); (3) CEAP clinical classification (C2–C6), independently assessed by two experienced vascular surgeons; (4) Venous Clinical Severity Score (VCSS), a validated 10-item instrument assessing the severity of chronic venous disease on a scale from 0 to 30, with higher scores indicating greater severity (19); (5) presence of venous symptoms including leg heaviness, aching, swelling, cramps, itching, and restless legs; (6) presence of complications such as superficial thrombophlebitis, lipodermatosclerosis, and venous ulceration; (7) comorbidities including hypertension, diabetes mellitus, obesity (BMI ≥ 30 kg/m^2^), osteoarthritis, and other chronic conditions, assessed using the Charlson Comorbidity Index (CCI) (20); and (8) current use of compression therapy (elastic compression stockings or bandages) and/or venoactive drug therapy.

#### 2.4.3 Psychological assessment

Psychological status was evaluated using the Hospital Anxiety and Depression Scale (HADS) (21), a well-validated 14-item self-report questionnaire comprising two subscales: anxiety (HADS-A) and depression (HADS-D), each containing 7 items scored from 0 to 3. Total subscale scores range from 0 to 21 for each dimension.

#### 2.4.4 Outcome measures: health-related quality of life assessment

HRQoL, the primary outcome of this study, was assessed using the Aberdeen Varicose Vein Questionnaire (AVVQ), a validated disease-specific instrument specifically designed to evaluate the impact of varicose veins on patients’ quality of life (22). The AVVQ is a 13-item self-administered questionnaire that encompasses multiple domains relevant to the experience of living with varicose veins, including pain and discomfort, ankle edema, the use of compression hosiery, presence of ulceration, cosmetic appearance, and the impact of varicose veins on daily and leisure activities. The total AVVQ score ranges from 0 to 100, with higher scores indicating a greater degree of impairment in quality of life.

### 2.5 Statistical analysis

All statistical analyses were performed using SPSS (version 26.0) and R (version 4.2.0), with a two-tailed *p* < 0.05 considered statistically significant. Variables with a normal distribution are expressed as mean ± standard deviation, while variables with a non-normal distribution are expressed as median (interquartile range). Categorical variables are summarized as frequency and percentage. Independent *t*-tests, one-way analysis of variance (ANOVA), Mann–Whitney U tests, and Pearson/Spearman correlation were used to assess univariate associations with the total AVVQ score. Variables with a *p*-value <0.05 in the univariate analysis were included in a multiple linear regression model using stepwise selection. Multicollinearity was examined using the variance inflation factor (VIF; VIF > 10 indicates severe multicollinearity).

### 2.6 Quality control

Several measures were implemented to ensure data quality and reliability: (1) all research assistants underwent standardized training prior to data collection regarding interview techniques, questionnaire administration, and data recording procedures; (2) clinical classification by CEAP was independently performed by two vascular surgeons, and any discrepancies were resolved through consensus with a third senior surgeon; (3) all completed questionnaires were reviewed for completeness and logical consistency on the day of collection, and any missing or ambiguous responses were clarified with the participants immediately; and (4) double data entry was performed to minimize transcription errors.

## 3 Results

### 3.1 General information

During the study period from December 2023 to April 2025, a total of 280 patients with lower extremity varicose veins were screened for eligibility. Of these, 24 patients were excluded: 8 did not meet the inclusion criteria (3 had secondary varicose veins, 3 had a history of previous surgical treatment, and 2 had severe systemic diseases), 10 declined to participate, and 6 returned incomplete questionnaires (completion rate <80%). Therefore, a final sample of 256 patients was included in the analysis, yielding an effective response rate of 91.4% (256/280). The sample size exceeded the minimum requirement of 210 participants calculated *a priori*. The baseline characteristics of the study population are presented in Table 1.

**Table 1:** Basic information of patients with lower extremity varicose veins (*N* = 256).

Variable	*n* (%) or Mean ± SD or Median (IQR)
Age (years)	
≤40	52 (20.3)
41–60	126 (49.2)
>60	78 (30.5)
Sex	
Male	100 (39.1)
Female	156 (60.9)
BMI (kg/m^2^)	
<25.0	108 (42.2)
25.0–29.9	106 (41.4)
≥30.0	42 (16.4)
Marital status	
Married	198 (77.3)
Single	28 (10.9)
Divorced	30 (11.7)
Educational level	
Primary school or below	62 (24.2)
Secondary/high school	112 (43.8)
College/university or above	82 (32.0)
Employment status	
Employed	148 (57.8)
Unemployed	38 (14.8)
Retired	70 (27.3)
Occupation type (among employed, *n* = 148)	
Sedentary	42 (28.4)
Prolonged standing	64 (43.2)
Mixed	42 (28.4)
Monthly household income (CNY)	
<5,000	78 (30.5)
5,000-10,000	108 (42.2)
>10,000	70 (27.3)
Current smoker	52 (20.3)
Drinking	
Yes	68 (26.6)
No	188 (73.4)
Regular physical activity	
Yes	86 (33.6)
No	170 (66.4)
Duration of varicose veins (years)	8.0 (4.0–15.0)
Laterality	
Unilateral	108 (42.2)
Bilateral	148 (57.8)
CEAP classification	
C2	82 (32.0)
C3	76 (29.7)
C4	58 (22.7)
C5	24 (9.4)
C6	16 (6.3)
VCSS	8.2 ± 4.5
Venous symptoms	
Leg heaviness	196 (76.6)
Aching	178 (69.5)
Swelling	142 (55.5)
Cramps	118 (46.1)
Itching	96 (37.5)
Restless legs	72 (28.1)
Number of symptoms	3.1 ± 1.6
Complications	
Superficial thrombophlebitis	32 (12.5)
Lipodermatosclerosis	38 (14.8)
Venous ulceration	16 (6.3)
Charlson Comorbidity Index	1.4 ± 1.2
Comorbidities	
Hypertension	82 (32.0)
Diabetes mellitus	36 (14.1)
Obesity (BMI ≥ 30 kg/m^2^)	42 (16.4)
Osteoarthritis	28 (10.9)
Compression therapy use	94 (36.7)
Venoactive drug therapy	76 (29.7)
HADS-Anxiety score	6.8 ± 3.9
HADS-Depression score	5.6 ± 3.7

CEAP, Clinical-Etiological-Anatomical-Pathophysiological; VCSS, Venous Clinical Severity Score; BMI, body mass index; IQR, interquartile range; SD, standard deviation.

### 3.2 Univariate analysis of factors associated with AVVQ scores

The mean total AVVQ score for the entire study population was 18.6 ± 9.4, indicating a moderate level of QoL impairment. Univariate analyses identified multiple factors significantly associated with the Aberdeen Varicose Vein Questionnaire (AVVQ) total score (Table 2). Statistically significant categorical variables included sex, BMI category, educational level, employment status, occupation type (among employed patients), monthly household income, regular physical activity, laterality (unilateral vs. bilateral), CEAP classification, presence of complications, and use of compression therapy. Among continuous variables, disease duration, VCSS, number of venous symptoms, Charlson Comorbidity Index, and HADS-Anxiety and HADS-Depression scores showed significant associations with the AVVQ score. In contrast, no significant associations were observed for age, marital status, smoking status, drinking, or venoactive drug therapy.

**Table 2:** Univariate analysis of factors associated with AVVQ total scores (*N* = 256).

Variable	AVVQ score (Mean ± SD)	Test statistic	*p* value
Sex		t = −3.92	<0.001*
Male	15.8 ± 8.2		
Female	20.4 ± 9.8		
Age group (years)		*F* = 2.14	0.120
≤40	17.2 ± 8.6		
41–60	18.4 ± 9.2		
>60	19.8 ± 10.1		
BMI category (kg/m^2^)		*F* = 8.76	<0.001*
<25.0	16.2 ± 8.4		
25.0–29.9	18.8 ± 9.0		
≥30.0	24.6 ± 10.2		
Marital status		*F* = 1.52	0.221
Married	18.2 ± 9.2		
Single	18.8 ± 9.6		
Divorced	20.8 ± 10.4		
Educational level		*F* = 6.32	0.002*
Primary school or below	21.8 ± 10.4		
Secondary/high school	18.6 ± 9.2		
College/university or above	15.8 ± 7.8		
Employment status		*F* = 4.92	0.008*
Employed	17.4 ± 8.8		
Unemployed	22.4 ± 10.6		
Retired	19.2 ± 9.4		
Occupation type (*n* = 148)		*F* = 5.96	0.003*
Sedentary	15.2 ± 7.6		
Prolonged standing	20.2 ± 9.4		
Mixed	16.8 ± 8.8		
Monthly household income		*F* = 5.24	0.006*
<5,000 CNY	21.4 ± 10.2		
5,000–10,000 CNY	18.2 ± 8.8		
>10,000 CNY	16.0 ± 8.4		
Smoking status		*F* = 0.86	0.424
Current smoker	17.6 ± 8.8		
Former smoker	19.4 ± 9.6		
Non-smoker	18.8 ± 9.6		
Drinking		t = 0.72	0.472
Yes	18.0 ± 8.6		
No	18.8 ± 9.6		
Regular physical activity		t = 3.28	0.001*
Yes	15.6 ± 7.8		
No	20.2 ± 9.8		
Disease duration		r = 0.32	<0.001*
Laterality		t = −3.64	<0.001*
Unilateral	16.0 ± 8.2		
Bilateral	20.6 ± 9.8		
CEAP classification		*F* = 24.86	<0.001*
C2	12.4 ± 6.2		
C3	16.8 ± 7.4		
C4	22.6 ± 8.8		
C5	27.4 ± 9.6		
C6	32.8 ± 10.2		
VCSS		r = 0.64	<0.001*
Number of symptoms		r = 0.56	<0.001*
Presence of complications		t = −5.42	<0.001*
No	16.4 ± 8.2		
Yes	25.2 ± 9.8		
Charlson Comorbidity Index		r = 0.28	<0.001*
Compression therapy use		t = 2.26	0.024*
Yes	16.8 ± 8.6		
No	19.6 ± 9.8		
Venoactive drug therapy		t = 1.32	0.186
Yes	17.6 ± 8.8		
No	19.0 ± 9.6		
HADS-Anxiety score		r = 0.52	<0.001*
HADS-Depression score		r = 0.48	<0.001*

AVVQ, Aberdeen Varicose Vein Questionnaire; BMI, body mass index; CEAP, Clinical-Etiological-Anatomical-Pathophysiological; VCSS, Venous Clinical Severity Score; HADS, Hospital Anxiety and Depression Scale; CNY, Chinese Yuan; SD, standard deviation. * Statistically significant (*P* < 0.05). t = independent *t*-test; F = one-way ANOVA; r = Pearson or Spearman correlation coefficient.

### 3.3 Multiple linear regression analysis

Variables significant in univariate analysis (*p* < 0.05) were entered into a stepwise multiple linear regression model. The final model was statistically significant (*F* = 51.24, *p* < 0.001) and explained 62.3% of the variance in AVVQ scores (adjusted *R*^2^ = 0.611). As shown in Table 3, eight variables were independently associated with the AVVQ total score: VCSS (*β* = 0.312, *p* < 0.001), HADS-Anxiety score (*β* = 0.224, *p* < 0.001), CEAP classification (*β* = 0.198, *p* < 0.001), HADS-Depression score (*β* = 0.156, *p* = 0.003), number of symptoms (*β* = 0.142, *p* = 0.005), BMI (*β* = 0.128, *p* = 0.008), regular physical activity (*β* = −0.108, *p* = 0.018), and bilateral involvement (*β* = 0.096, *p* = 0.032). Variance inflation factor values ranged from 1.12 to 2.68, indicating no substantial multicollinearity. These findings suggest that greater clinical severity, higher anxiety and depression levels, more venous symptoms, higher BMI, absence of regular physical activity, and bilateral disease are independent predictors of worse HRQoL in patients with lower extremity varicose veins.

**Table 3:** Multiple linear regression analysis of factors independently associated with AVVQ total scores (*N* = 256).

Variable	B	SE	*β*	95% CI	*t*	*P* value	VIF
Constant	−4.826	2.412	–	−9.580 to −0.072	−2.00	0.047	–
VCSS	0.652	0.084	0.312	0.486 to 0.818	7.76	<0.001	2.14
HADS-A score	0.538	0.092	0.224	0.356 to 0.720	5.85	<0.001	1.86
CEAP classification	2.846	0.620	0.198	1.624 to 4.068	4.59	<0.001	2.68
HADS-D score	0.396	0.132	0.156	0.138 to 0.654	3.00	0.003	1.72
Number of symptoms	0.832	0.293	0.142	0.256 to 1.408	2.84	0.005	1.94
BMI (kg/m^2^)	0.334	0.125	0.128	0.088 to 0.580	2.67	0.008	1.32
Regular physical activity (ref: no)	−2.164	0.910	−0.108	−3.952 to −0.376	−2.38	0.018	1.12
Bilateral involvement (ref: unilateral)	1.826	0.848	0.096	0.158 to 3.494	2.15	0.032	1.48

Model summary: *R*^2^ = 0.623; Adjusted *R*^2^ = 0.611; *F* = 51.24; *p* < 0.001. AVVQ, Aberdeen Varicose Vein Questionnaire; B, unstandardized regression coefficient; SE, standard error; *β*, standardized regression coefficient; CI, confidence interval; VIF, variance inflation factor; VCSS, Venous Clinical Severity Score; HADS-A, Hospital Anxiety and Depression Scale–Anxiety; HADS-D, Hospital Anxiety and Depression Scale–Depression; CEAP, Clinical-Etiological-Anatomical-Pathophysiological; BMI, body mass index.

## 4 Discussion

This cross-sectional study comprehensively investigated the factors influencing HRQoL in 256 patients with LEVV using a validated disease-specific instrument, the AVVQ. The mean AVVQ score of 18.6 ± 9.4 indicated a moderate level of QoL impairment in this population, which is consistent with findings from previous international studies (23, 24). The multiple linear regression model identified eight independent predictors that collectively explained 62.3% of the variance in AVVQ scores, underscoring the multifactorial nature of QoL impairment in LEVV patients. These predictors encompassed clinical severity measures (VCSS, CEAP classification, number of symptoms, and bilateral involvement), psychological factors (anxiety and depression), a modifiable metabolic parameter (BMI), and a behavioral factor (regular physical activity). The substantial proportion of variance explained by this model suggests that these factors capture the core determinants of disease-specific QoL in this patient population and provides a robust foundation for developing targeted interventions.

The Venous Clinical Severity Score (VCSS) emerged as the strongest independent predictor of AVVQ scores (*β* = 0.312), followed by CEAP classification (*β* = 0.198) and the number of venous symptoms (*β* = 0.142). These findings collectively emphasize the central role of clinical disease severity in determining HRQoL in patients with LEVV. The strong positive correlation between VCSS and AVVQ scores (r = 0.64 in univariate analysis) aligns with prior studies demonstrating that VCSS is a reliable predictor of patient-reported outcomes in chronic venous disease (25). Vasquez et al. (19) validated the revised VCSS as a comprehensive measure of venous disease severity that correlates well with both generic and disease-specific QoL instruments, and our findings further support its utility in clinical practice for identifying patients with significant QoL impairment.

The significant association between CEAP classification and AVVQ scores observed in our study is consistent with the findings of Kahn et al. (14), who reported a progressive deterioration in QoL across CEAP classes in an international cohort of patients with chronic venous disease. In our cohort, AVVQ scores increased markedly from C2 (12.4 ± 6.2) to C6 (32.8 ± 10.2), representing a nearly threefold increase in QoL impairment. This stepwise deterioration reflects the cumulative impact of disease progression on patients’ physical functioning, pain, and daily activities. Notably, patients classified as C4 and above demonstrated substantially higher AVVQ scores, suggesting that the development of skin changes represents a critical threshold beyond which QoL deterioration accelerates. This observation has important clinical implications, as it supports the rationale for early intervention before the onset of irreversible dermatological complications such as lipodermatosclerosis and venous ulceration (7).

The number of venous symptoms was also independently associated with impaired quality of life. This finding is consistent with the studies by Carpentier et al. (9) and Zenati et al. (13), who demonstrated that symptom burden is a key driver of worsening quality of life in chronic venous disease. The cumulative effect of multiple symptoms, including leg heaviness, aching, swelling, and cramps, may exert a synergistic impact on patients’ functional capacity, sleep quality, and overall well-being that extends beyond the influence of individual symptoms. This underscores the importance of comprehensive symptom assessment and multimodal symptom management strategies in clinical practice.

A notable finding of this study was the independent and significant association of both anxiety and depression with QoL impairment, even after adjustment for clinical severity measures. These findings are consistent with a growing body of evidence indicating a bidirectional relationship between chronic venous disease and mental health. Sritharan et al. (10) reported that patients with symptomatic varicose veins had a significantly higher prevalence of depression compared to the general population, and depression was independently associated with poorer quality of life outcomes. Similarly, a systematic review by Zenati et al. (13) identified psychological distress as a consistent predictor of impaired HRQoL in studies on chronic venous disease. The mechanisms underlying this association are likely multifactorial. On one hand, the chronic nature of venous symptoms, particularly pain, heaviness, and swelling, may contribute to the development of anxiety and depressive symptoms through persistent discomfort and functional limitations. On the other hand, the visible cosmetic disfigurement associated with varicose veins may lead to body image dissatisfaction, social embarrassment, and avoidance behaviors, particularly among female patients, who constituted 60.9% of the study population in this research (10, 11). Furthermore, psychological distress may amplify the perception and reporting of physical symptoms, creating a self-reinforcing cycle of symptom burden and emotional distress, thereby perpetuating a decline in quality of life (26).

BMI was identified as an independent predictor of worse QoL, with obese patients demonstrating significantly higher AVVQ scores compared to those with normal weight. This finding is consistent with previous studies that have established obesity as both a risk factor for the development and progression of varicose veins and a determinant of QoL impairment in patients with chronic venous disease (6, 27). The detrimental impact of elevated BMI on venous QoL is likely mediated through multiple pathways. Increased intra-abdominal pressure associated with central adiposity impedes venous return, exacerbates venous hypertension, and promotes venous reflux, thereby worsening the hemodynamic derangements underlying varicose veins (28). Additionally, obesity is associated with a chronic pro-inflammatory state characterized by elevated levels of interleukin-6, tumor necrosis factor-*α*, and C-reactive protein, which may accelerate venous wall remodeling and valvular deterioration (29). From a functional perspective, excess body weight increases the mechanical load on the lower extremities, potentiating symptoms such as leg heaviness, fatigue, and swelling, while simultaneously limiting patients’ mobility and capacity for physical activity. These findings highlight the importance of weight management counseling as an integral component of the treatment plan for overweight and obese patients with LEVV.

Regular physical activity emerged as the only modifiable behavioral factor independently associated with improved quality of life in the regression model. Patients who engaged in at least 150 min of moderate-intensity aerobic exercise per week reported significantly lower AVVQ scores. The protective effect of regular exercise is biologically plausible, as physical activity enhances calf muscle pump function, promotes venous return in the lower limbs, thereby reducing ambulatory venous pressure and mitigating the hemodynamic consequences of venous reflux (30). Furthermore, regular exercise has well-recognized benefits for weight management, cardiovascular health, and mental well-being, all of which may indirectly contribute to quality of life improvement in patients with LEVV (31). Notably, only 33.6% of patients in our cohort met the World Health Organization-recommended threshold for regular physical activity, indicating that a substantial proportion of LEVV patients may benefit from structured exercise counseling and prescription.

Bilateral varicose vein involvement was independently associated with higher AVVQ scores compared to unilateral disease. This finding, while intuitive, has received relatively limited attention in the existing literature. Bilateral involvement doubles the anatomical burden of disease, potentially exacerbates symptoms such as swelling and heaviness in both lower extremities simultaneously, and may have a greater impact on functional capacity and mobility than unilateral disease. Moreover, the cosmetic impact of bilateral varicose veins may contribute to greater psychological distress and body image dissatisfaction. In our cohort, 57.8% of patients had bilateral disease, which is consistent with the prevalence reported in other hospital-based studies (32).

## 5 Limitation

However, several limitations should be acknowledged. First, the cross-sectional design precludes the establishment of causal relationships between the identified predictors and QoL outcomes. Moreover, it does not allow us to determine which factors actually drove patients to seek medical treatment. Longitudinal studies are needed to confirm the directionality of associations, to evaluate changes in QoL over time, and to explore the determinants of treatment-seeking decisions in patients with lower extremity varicose veins. Second, the study was conducted at a single tertiary care center, which may limit the generalizability of the findings to patients managed in primary care or community settings, as well as to populations in other regions or countries with different demographic characteristics, healthcare systems, and cultural contexts.

## 6 Conclusion

In conclusion, this study demonstrates that QoL in patients with lower extremity varicose veins is significantly influenced by a combination of clinical, psychological, anthropometric, and behavioral factors. The VCSS, anxiety, CEAP classification, depression, number of venous symptoms, BMI, absence of regular physical activity, and bilateral involvement were identified as independent predictors of worse disease-specific QoL. These findings underscore the importance of adopting a comprehensive, multidimensional approach to the assessment and management of LEVV patients that addresses not only the hemodynamic and clinical aspects of the disease but also the psychological and lifestyle factors that contribute substantially to the overall disease burden. Future prospective and interventional studies are warranted to validate these findings and to evaluate the effectiveness of integrated management strategies incorporating psychological support, exercise prescription, and weight management on QoL outcomes in this patient population.

## Data Availability

The original contributions presented in the study are included in the article/supplementary material, further inquiries can be directed to the corresponding authors.
